# Supplementary material for: The beneficial effect of physical activity on cognitive function in community-dwelling older persons with locomotive syndrome
Source: PeerJ. 2021 Oct 15;9:e12292. doi: 10.7717/peerj.12292 (PMC8522643; doi:10.7717/peerj.12292)
Supplement: Supplemental Information 2 [file peerj-09-12292-s002.pdf]

Supplementary Table S1

Sample of Addenbrooke's Cognitive Examination-Revised (ACE-R) (*Mioshi et al., 2006; Hashimoto, 2010*)

Orientation/Attention	[Total score 0–18]
Orientation	[Score 0–10]
Registration<br>➢ Say: "I'm going to give you three words and I'd like you to repeat after me: 'lemon, key and ball'. After the subject repeats the words, say, 'Try to remember them because I'm going to ask you later.'" Score only the first trial (repeat three times if necessary).<br>Register the number of trials	[Score 0–3]
Attention & Concentration	[Score 0–5]
Memory	[Total score 0–26]
Recall-(1)<br>➢ Ask: "Which 3 words did I ask you to repeat and remember?"	[Score 0–3]
Anterograde Memory	[Score 0–7]
Retrograde Memory	[Score 0–4]
Recall (2)	[Score 0–7]
Recognition	[Score 0–5]
Verbal Fluency	[Total score 0–14]
Letter 'P' and animal	
➢ Letters<br>Say: "I'm going to give you a letter of the alphabet and I'd like you to generate as many words as you can beginning with that letter, but not names of people or places. Are you ready? You've got 1 minute and the letter is 'P'"	[Score 0–7]
➢ Animals<br>Say: "Now, can you name as many animals as possible, beginning with any letter?"	[Score 0–7]
Language	[Total score 0–26]
Comprehension	
➢ Show written instruction:<br>"Close your eyes"	[Score 0–1]
➢ Three-stage command:<br>"Take the paper in your right hand. Fold the paper in half. Put the paper on the floor."	[Score 0–1]
Writing	[Score 0–1]
Repetition	[Score 0–6]
Naming	[Score 0–12]
Comprehension	[Score 0–4]
Reading	[Score 0–1]

Visuospatial	[Total score 0–16]
Abilities	
➢ Overlapping pentagons: Ask the subject to copy this diagram:	[Score 0–1]
➢ Wire cube: Ask the subject to copy this drawing (for scoring, see the instruction guide)	[Score 0–2]
➢ Clock: Ask the subject to draw a clock face with numbers and the hands at ten past five. (for scoring, see the instruction guide: circle = 1, numbers = 2, hands = 2 if all	[Score 0–5]
correct)	[Score 0–4]
➢ Ask the subject to count the dots without pointing to them	[Score 0–4]
➢ Ask the subject to identify the letters	
